# Performance enhancement and PAPR reduction for MIMO based QAM-FBMC systems

**DOI:** 10.1371/journal.pone.0296999

**Published:** 2024-01-11

**Authors:** Emad S. Hassan

**Affiliations:** 1 Department of Electrical Engineering, College of Engineering, Jazan University, Jizan, Saudi Arabia; 2 Department of Electronics and Electrical Communications, Faculty of Electronic Engineering, Menoufia University, Menouf, Egypt; Thamar University: Dhamar University, YEMEN

## Abstract

Filter Bank Multi-Carrier (FBMC) is attracting significant interest as a multi-carrier modulation (MCM) approach for future communication systems. It offers numerous advantages in contrast to Orthogonal Frequency Division Multiplexing (OFDM). Nonetheless, similar to many other MCM techniques, FBMC encounters a significant challenge with a high Peak-to-Average Power Ratio (PAPR). Additionally, incorporating Multiple-Input and Multiple-Output (MIMO) into FBMC presents heightened difficulties due to the presence of complex interference and increased computational complexity. In this paper, we first study the performance analysis of MIMO based Quadrature Amplitude Modulation (QAM)-FBMC systems considering the system complexity and interference. To enhance coverage effectively using beamforming with multiple antennas, it is essential to reduce PAPR to minimize the input backoff (IBO) required by nonlinear power amplifiers. Therefore, we propose new PAPR reduction method for MIMO based QAM-FBMC systems leveraging the null space within the MIMO channel using clipping and filtering (CF) technique. The PAPR reduction signals generated in this process are then mapped to the null space of the overall MIMO channel for each frequency block. Through computer simulations using a nonlinear power amplifier model, we illustrate that the proposed method substantially enhances both PAPR and throughput of MIMO based FBMC systems compared to conventional methods.

## 1. Introduction

Compared to single carrier modulation, multi-carrier modulations have garnered greater attention due to their ability to effectively handle frequency-selective fading channels [1–3]. As such, multi-carrier modulation, especially Orthogonal Frequency Division Multiplexing (OFDM), is a promising candidate for future communication systems. OFDM, which avoids both inter-symbol interference and inter-channel interference, has been widely adopted; however, it is not without its limitations.

OFDM-based systems are sensitive to timing offsets caused by imperfect synchronization and rapidly changing radio channel conditions [4]. Additionally, high side-lobe levels in the filters’ frequency response make OFDM susceptible to carrier frequency offsets (CFO), leading to inter-carrier interference (ICI) and performance degradation [5]. Moreover, the insertion of cyclic prefixes (CP) in OFDM-based networks reduces their spectral efficiency [6]. Recently, accurate estimation of carrier and timing offset, as well as the channel, is feasible in OFDM-based systems with a significant potential for parallel processing [7, 8] but this at the cost of system complexity. Also, the suppression of side-lobes can be achieved through the utilization of a transmit filter (pulse shaping filter) [7, 8].

As an alternative to OFDM, Filter Bank Multi-Carrier (FBMC) has emerged as a technique to overcome the limitations of OFDM. FBMC applies a well-designed prototype filter to each subcarrier, in contrast to CP-OFDM, which employs filtering for the entire frequency band, leading to a sinc-shaped spectrum [6]. This design makes FBMC less sensitive to ICI and more efficient in terms of spectral efficiency, as it eliminates the need for CP [6]. Additionally, FBMC’s modulation prototype filters provide better immunity to CFO, improving its performance [4]. FBMC has also proven to be an effective tool in spectrum sensing compared to the Fast Fourier Transform (FFT) [9]. Comparisons of spectral efficiency between FBMC and OFDM-based CR networks have shown that FBMC outperforms OFDM [10]. Therefore, FBMC has many applications especially in wireless sensor networks and internet-of-things [11–15].

While FBMC offers various advantages over OFDM, applying certain techniques to FBMC, such as Multiple-Input and Multiple-Output (MIMO), becomes more challenging due to the presence of complex interference [15]. Some research has combined MIMO with FBMC-based systems, but this often comes at the cost of increased computational complexity and reduced spectral efficiency [16–20]. In this paper, we propose a novel MIMO-based FBMC transceiver that utilizes Quadrature Amplitude Modulation (QAM) transmission to address interference issues commonly found in conventional Orthogonal Quadrature Amplitude Modulation (OQAM)-FBMC systems.

High Peak-to-Average Power Ratio (PAPR) is a significant challenge in multi-carrier modulation (MCM) techniques [1, 19, 20], including FBMC. High PAPR can degrade system performance, particularly in terms of bit-error rate (BER). The unique structure of FBMC, with its overlapping subcarriers, makes applying traditional PAPR reduction techniques designed for OFDM signals impractical [1, 19, 20].

Several publications have explored PAPR reduction in FBMC-based systems, using methods such as clipping, companding [21], and selective mapping [22–24]. However, many of these techniques suffer from high computational complexity. Furthermore, none of the previous works have investigated PAPR reduction in MIMO-based QAM-FBMC systems.

In summary, this paper makes a two-fold contribution: (1) introducing a novel MIMO-enabled FBMC transceiver that employs QAM transmission to effectively mitigate interference challenges experienced in traditional OQAM-FBMC systems; (2) presenting a method for reducing PAPR in MIMO-based FBMC systems through transmitter-side signal processing, leveraging the null space within the MIMO channel. These PAPR reduction signals are projected onto the null space of the integrated MIMO channel. As the MIMO channel varies for each frequency block, the PAPR reduction signals are tailored to each frequency block’s null space. After an iterative process, these PAPR reduction signals are collectively transmitted by the transmitter.

The subsequent sections of the paper are structured as follows: Section 2 summarizes the related work. Section 3 provides an overview of the system model. Section 4 outlines the proposed PAPR reduction method. Section 5 presents numerical results obtained from computer simulations. Finally, Section 6 serves as the paper’s conclusion and outlines potential future work.

## 2. Related work

The quest for technological advancements has paved the way for a transformative era, emphasizing the importance of employing suitable modulation techniques and compatible devices. This pursuit seeks to usher in a new age that surpasses previous technological achievements. Central to this endeavor is the development of a waveform characterized by high spectral efficiency and minimal PAPR. Within this context, the work in [4] delves into the comparative analysis of three prominent multicarrier modulation techniques: OFDM and OQAM-FBMC. The evaluation criteria encompass spectral efficiency, BER, and PAPR, utilizing various subcarriers and modulation techniques. The findings distinctly highlight the limitations of OFDM, particularly in terms of spectral efficiency, exacerbated by the presence of CP. In contrast, FBMC emerges as highly functional alternative. However, this approach introduces an increase in PAPR.

The authors in [5] investigate a response difference phenomenon observed during the design of multi-tap equalizers for FBMC/OQAM based massive MIMO systems, which negatively impacts equalization performance. In response to this challenge, the authors propose a multi-stage equalization-based design scheme for multi-tap equalizers, characterized by low implementation complexity. This scheme effectively mitigates the identified phenomenon, leading to a substantial improvement in equalization performance. The study in [6] conducts a comparative analysis of FBMC and OFDM systems in multipath fading channels, focusing on their theoretical system capacities. While OFDM exhibits resistance to inter-symbol interference (ISI), it generates high levels of out-of-band (OOB) radiation. In contrast, FBMC, with subcarrier-wise filtering, mitigates OOB radiation and reduces adjacent channel interference (ACI) but is susceptible to ISI and ICI due to multipath fading. The key objective is to evaluate the theoretical system capacities of FBMC and OFDM, considering ACI and multipath fading in an asynchronous scenario.

In the realm of 5th generation (5G) wireless telecommunications, OFDM with OQAM has recently attracted considerable attention. The research outlined in [7] showcases the generation of OFDM-OQAM through the Hilbert transform, aligning it with single sideband modulation (SSB) with roots in analog telecommunications. The transmit filter is characterized by a complex structure, encompassing the root raised cosine spectrum and the Hilbert transform. Additionally, [8] addresses the improvement of data detection in single-user massive MIMO through re-transmissions and enhances BER using turbo codes.

Despite the advantages of FBMC, like other multicarrier waveforms, it grapples with a high PAPR issue. Therefore, several publications have delved into the realm of PAPR reduction within FBMC-based systems. The work presented in [9] explores the application of logarithmic rooting companding for PAPR reduction in FBMC- OQAM systems, comparing its performance with other nonlinear companding techniques such as *μ*-law, A-law, rooting, and tangent rooting, commonly used for PAPR reduction in FBMC-OQAM systems. In [21], the reduction of PAPR is achieved through the application of clipping and companding methods.

Traditional PAPR reduction schemes designed for OFDM signals are not directly applicable to FBMC-OQAM due to its overlapping structure. Therefore, the authors in [22] introduce Dispersive Selective Mapping (DSLM), an extended and generalized version of SLM, as a proposed solution. In [23] they introduce a Trellis-Based SLM (TSLM) scheme with dynamic programming, exploring optimal phase rotation vectors. Additionally, the investigation into PAPR reduction continues with a novel tone reservation (TR) scheme considering the overlapping nature of FBMC-OQAM symbols in [24].

Nevertheless, numerous existing techniques in the literature encounter challenges related to high computational complexity. Additionally, prior research has not delved into the realm of PAPR reduction specifically tailored for MIMO based QAM-FBMC systems. These gaps underscore the significance of the method proposed in this work, addressing both computational complexity concerns and the unique requirements of MIMO-based QAM-FBMC systems.

## 3. MIMO based QAM-FBMC system model

As discussed earlier, OQAM-FBMC presents several advantages over CP-OFDM, albeit with the inherent challenge of intrinsic interference. This interference complicates the design of MIMO transceivers and channel estimation. The literature has proposed various techniques to mitigate the impact of intrinsic interference in conventional OQAM-FBMC. For instance, some approaches involve spreading symbols in time or frequency [25], while others focus on using QAM transmission [26, 27].

In this section, we introduce a novel MIMO transceiver designed for the QAM-FBMC system. QAM-FBMC, in comparison to OQAM-FBMC, exhibits several distinctive features and design variances. It employs multiple prototype filters and supports complex-valued QAM symbols [28], whereas conventional OQAM-FBMC utilizes a single prototype filter and conveys solely real-valued data. Another significant divergence is in the pulse-shape filtering of subcarriers: OQAM-FBMC employs non-rectangular pulse-shape filtering per subcarrier, eliminating spectral redundancy but at the cost of loosening the orthogonality condition in the real domain. In contrast, the presented QAM-FBMC system separates adjacent subcarriers using at least two filter banks, maintaining near orthogonality in the complex domain.

**Fig 1:**
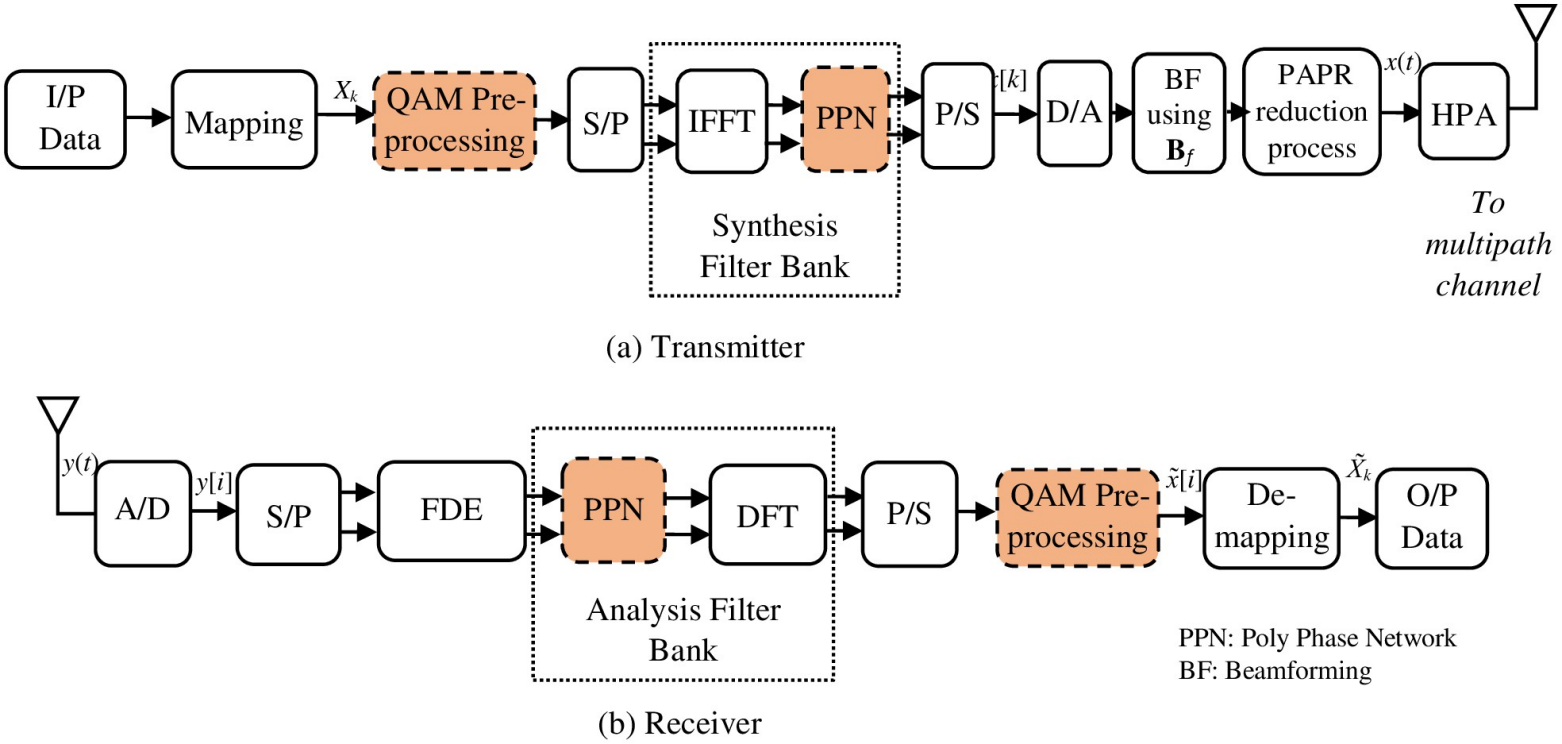
The typical QAM-FBMC transceiver structure.

**Fig 2:**
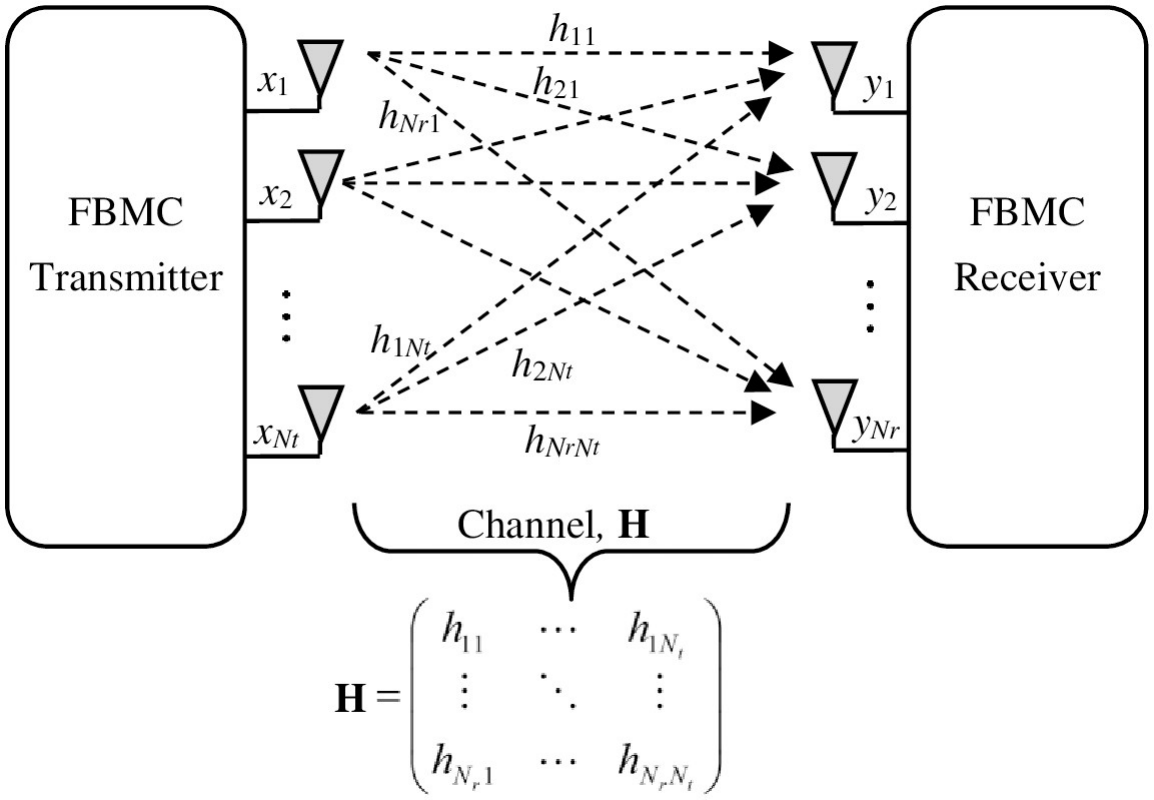
MIMO based QAM-FBMC transceiver structure.

Fig 1 illustrates the block diagram of a standard QAM-FBMC transceiver featuring *M* sub-carriers. While the MIMO based FBMC transceiver is shown in Fig 2 considering *N*_*t*_ transmitting antennas and *N*_*R*_ receiving antennas. We consider the case where *N*_*t*_ = *N*_*R*_ = *N*. Each of the *M* complex valued QAM symbols is modulated by a pulse shaping filter, *p*[*i*]. According to Fig 1, the transmit signal at the *i*th antenna is given as [3]:

$$x[n]=\sum_{m=1}^{M-1}\sum_{k\in Z}D_{m,k}p[n-kM/2]e^{j\frac{2\pi m}{M}(n-\frac{d}{2})e^{j\varphi_{m,k}}}$$ (1)

where *D*_*m*,*k*_ represents the real or imaginary section of QAM data using *mt*^*h*^ subcarrier and *k*^*th*^ transmitted symbol, *M* represents the subcarriers number and *d* is the amount of delay that depends on pulse shaping filter length. In this context, we assume that *p*[*n* − *kM*/2] is the same across all antennas.

In Eq (1), the data term *D*_*m*,*k*_ must be phase adjusted with a phase term, $e^{j\varphi_{m,k}}$, with $\varphi_{m,k}=\frac{\pi }{2}(m+k)$ to achieve the filters orthogonality conditions and to prevent interference among data symbols. The transmit signal, *x*[*n*] in (1) can be simplified as:

$$x[n]=\sum_{m=1}^{M-1}\sum_{k=0}^{\infty }D_{m,k}p_{m,k}\left[n\right]$$ (2)

The *m*-th transmitted data vector of length *L* = *lM* before beamforming (BF) stage can be rewritten as:

$$x\left[k\right]=W^{H}PD\left[k\right]$$ (3)

where *l* represents the overlapping coefficient, **W**^*H*^ is the *K*-point IDFT matrix and **P** represents the frequency domain filter coefficients matrix.

We assume that there exist *F* frequency blocks, each characterized by distinct channel conditions. In each frequency block, there are *S* = *M*/*F* subcarriers. In the context of frequency block *f* (*f* = 1, …, *F*), the channel matrix with dimensions *N* × *N* is **H** as indicated in Fig 2. We also assume that the transmitter possesses advance knowledge of all the channel matrices.

The transmitter produces the *N*-dimensional transmission signal vector **x**_*f*_[*k*], resulting from the multiplication of data vector in (3) by the *N* × *N* beamforming matrix **B**_*f*_. In this study, **B**_*f*_ is specifically derived using zero forcing with respect to the channel matrix **H**. Consequently, **x**_*f*_[*k*] is written as follows [3, 17]:

$$x_{f}\left[k\right]=B_{f}x\left[k\right]=\sqrt{P}H^{-1}x\left[k\right]$$ (4)

where $\sqrt{P}$ is used to achieve maximum transmission power constraint and **H**^-1^ is the zero forcing BF matrix.

Let **R** be the PAPR reduction matrix generated at the transmitter. Then the transmitted signal matrix after PAPR reduction step is **x**_*f*_ + **R** with size *N* × *S*. At the receiver side the received signal matrix with size *N* × *S* is written as [3]:

$$Y=H\left(x_{f}+R\right)+N_{r}$$ (5)

where **N**_*r*_ is the frequency-domain representation of noise matrix at receiver side.

The primary goal of the proposed PAPR reduction method is to achieve the necessary reduction in PAPR, while simultaneously mitigating the interference component (**HR**) originating from the PAPR reduction step as shown in Eq (5). It is important to note that the explanation above assumes that the transmission signal undergoes linear power amplification. This assumption is made because the PAPR reduction, as outlined in Section 4, is designed based on the premise of linear power amplification. In Section 5, we assess the performance of the proposed method by simulating scenarios involving nonlinear power amplification.

## 4. The proposed PAPR reduction method

This method entails conducting PAPR reduction signal processing at the transmitter side within the system model depicted in Fig 1. More precisely, it employs an adaptive PAPR reduction approach that leveraging the null space of the comprehensive channel, encompassing all links. This channel structure varies for each frequency block. The result is the generation of a PAPR reduction signal matrix, denoted as **R** as indicated by (5). This matrix serves to decrease PAPR while concurrently mitigating interference within the data stream, particularly at the user equipment (UE) receivers.

In the realm of PAPR reduction methods that leverage the null space in the MIMO channel, the literature introduces two techniques: clipping and filtering (CF) with channel-null constraint (CFCNC) [29, 30] and peak cancellation (PC) signal with the channel-null constraint (PCCNC) [31]. In this context, we adopt the CFCNC approach.

In the upcoming explanation, we will delve into the proposed method, with a particular emphasis on signal processing within the *f*-th frequency block. It’s worth noting that the same set of processes is applied across all *F* frequency blocks. During the *j*-th iteration (*j* = 1, …, *J* where *J* represents the no. of iterations), we first generate the matrix **R**^(*j*)^. At this point, we consider the transmission signal matrix at the *j*-th iteration, represented as **x**_***f***_ **+ R**^(*j*)^. Consequently, the estimated received signal matrix can be represented as:

$${\tilde{Y}}^{\left(j\right)}=H\left(x_{f}+R^{\left(j\right)}\right)$$ (6)

The time-domain received signal vector at antenna *r* (*r =* 1, …, *N*) is expressed as:

$$\left[{\tilde{y}}_{1}^{\left(j\right)}\ldots {\tilde{y}}_{N}^{\left(j\right)}\right]=W^{H}{\left[{\tilde{Y}}_{1}^{\left(j\right)}\ldots {\tilde{Y}}_{F}^{\left(j\right)}\right]}^{T}$$ (7)

The PAPR reduction signal vector in the time domain, denoted as $v_{r}^{\left(j\right)}$ for antenna *r*, is derived by applying a CF operation to ${\tilde{y}}_{r}^{\left(j\right)}$ to mitigate PAPR. The frequency-domain PAPR reduction signal matrix, with dimensions *N* × *S*, for the specific frequency block *f*, can be represented as follows:

$$\left[{\tilde{R}}_{1}^{\left(j\right)}\ldots {\tilde{R}}_{F}^{\left(j\right)}\right]={\left[{\tilde{v}}_{1}^{\left(j\right)}\ldots {\tilde{v}}_{N}^{\left(j\right)}\right]}^{T}\;W^{T}$$ (8)

where **W** represents the *N*-point DFT matrix.

Note, one important difference between the OFDM and the FBMC is that there is no overlapping in the time domain among the symbols of the OFDM, while for FBMC the symbols are overlapping over several neighboring symbols. This difference makes the application of iterative algorithms such as SLM or partial transmit sequence (PTS) is much more complicated in FBMC based systems than OFDM. Therefore, we considered CF method for PAPR reduction.

Let’s contemplate a downlink multiuser MIMO scenario in which we spatially multiplex *N*_*u*_ users, with each user having a single receiver antenna. In this context, we assume a massive MIMO environment, where we have a configuration with *N* > *N*_*u*_. The interference term (***H*R**) originating from the PAPR reduction step at UE can be removed by projecting this term onto the null space in overall *N* × *N* MIMO channel **H**. Since *N* > *N*_*u*_ we have *N* × *N- N*_*u*_ matrix **V** representing the null space in **H**. Therefore, **HV** = **O** (zero matrix). It is assumed that all column vectors of **V** are orthonormal. Hence, the presented method diminishes the PAPR through signal processing. Simultaneously, it effectively mitigates interference within the data stream as received by the UE receivers.

## 5. Numerical results and discussions

### A. simulation parameters

To assess the efficiency of the proposed PAPR reduction method, we conducted computer simulations with the key parameters outlined as follows. *N* = 50, *N*_*u*_ = 4 and the system employs *K* = 512 subcarriers, with the parameter *F* being subject to variation. To ensure precise measurement of PAPR levels the *M*-point DFT is 2048, resulting in a four-times oversampling factor. For the sake of generality in our evaluations, we made the assumption that the symbol constellation for each subcarrier follows an independent standard complex Gaussian distribution. Our approach involved implementing zero-forcing beamforming. The simulation parameters are summarized in Table 1.

**Table 1:** Parameters used in simulations.

Parameter	Value
*N*	50
*N*_*u*_	4
*K*	512
*M*	2048
*L*	4
*J*	20
BF	Zero-forcing
Channel model	Frequency selective fading

In simulating the non-linear power amplifiers at transmitter, we adopted the solid-state power amplifier (SSPA) model [32].


$$y\left(t\right)=G_{0}\;\frac{x(t)}{{\left(1+{\left(\left|x(t)\right|/A_{Sat}\right)}^{2c}\right)}^{1/2c}}$$ (9)

In Eq (9), we represent the input and output signals at time *t* as *x*(*t*) and *y*(*t*), respectively. Within this context, *G*_0_ corresponds to the amplification gain in the linear amplifier domain, while *A*_*Sat*_ denotes the saturation value for input amplitude. The parameter *c* regulates the smoothness of the transition from the linear to the saturation region. IBO, defined as the ratio of |*A*_*Sat*_|^2^ to the average signal power across all transmission antennas, is a critical metric. When the transmission signal enters the nonlinear region, it introduces interference signals both within and outside the signal bandwidth. For our evaluation, we set *G*_0_ to 1 and *A*_*Sat*_ to 0.05. The parameter *c* was established at 2.0, as referenced in [33].

For our channel model, we adopt frequency selective fading model [3–5], which is uncorrelated among transmitter-receiver antenna pairs and across different frequency blocks. The PAPR is a metric that represents the ratio of the maximum signal power to the mean signal power, it can be expressed as:

$$PAPR=\frac{P_{peak}}{P_{avg}}=\frac{max\left\{{\left|x(k)\right|}^{2}\right\}}{E\left\{{\left|x(k)\right|}^{2}\right\}}$$ (10)

where *E*[.] is the mean value. The complementary cumulative distribution function (CCDF) represents the probability that the PAPR exceeds a threshold value PAPR_0_ and is expressed as:

$$CCDF=\text{Pr}(PAPR(x(k))>{PAPR}_{0})$$ (11)

The power thresholds, T_h_ used in the clipping and filtering process to generate PAPR reduction signals is the signal power thresholds normalized by the signal power per antenna averaged over multiple channel realizations. To evaluate the cumulative throughput of *N*_*u*_ users, we employ the Shannon formula while considering the Bussgang theorem [34]. This calculation provides an upper bound on the achievable transmission rate, assuming ideal channel coding, without any errors.

### B. simulation results

**Fig 3:**
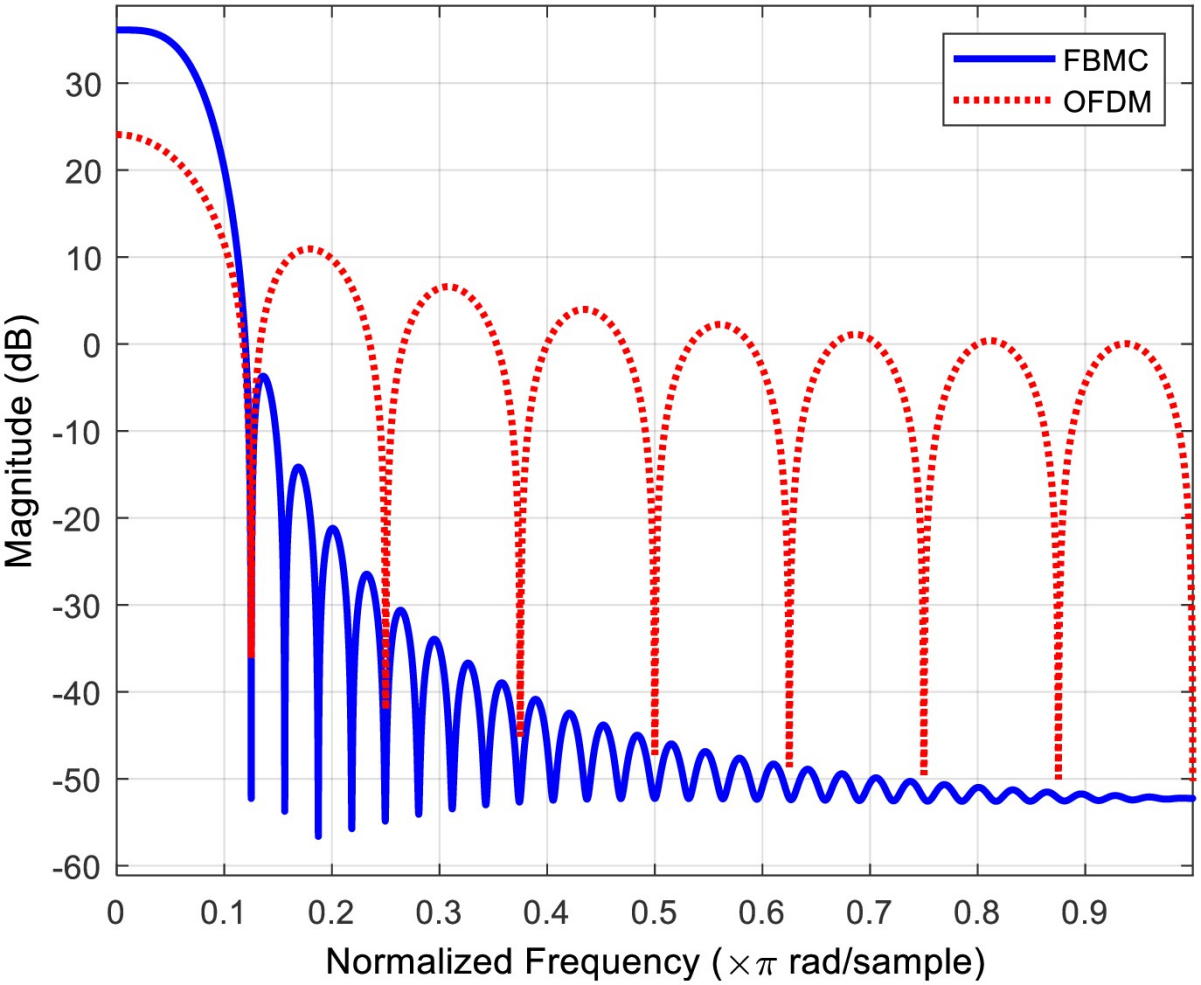
Power spectral density comparison of FBMC and OFDM signals.

The power spectral density (PSD) of both FBMC and OFDM signals is depicted in Fig 3. It’s evident that the OOB radiation of FBMC is significantly lower when compared to OFDM where FBMC uses pulse shaping filter which eliminates the out-of-band radiation. Therefore, FBMC is less susceptible to ICI and has higher spectral efficiency.

**Fig 4:**
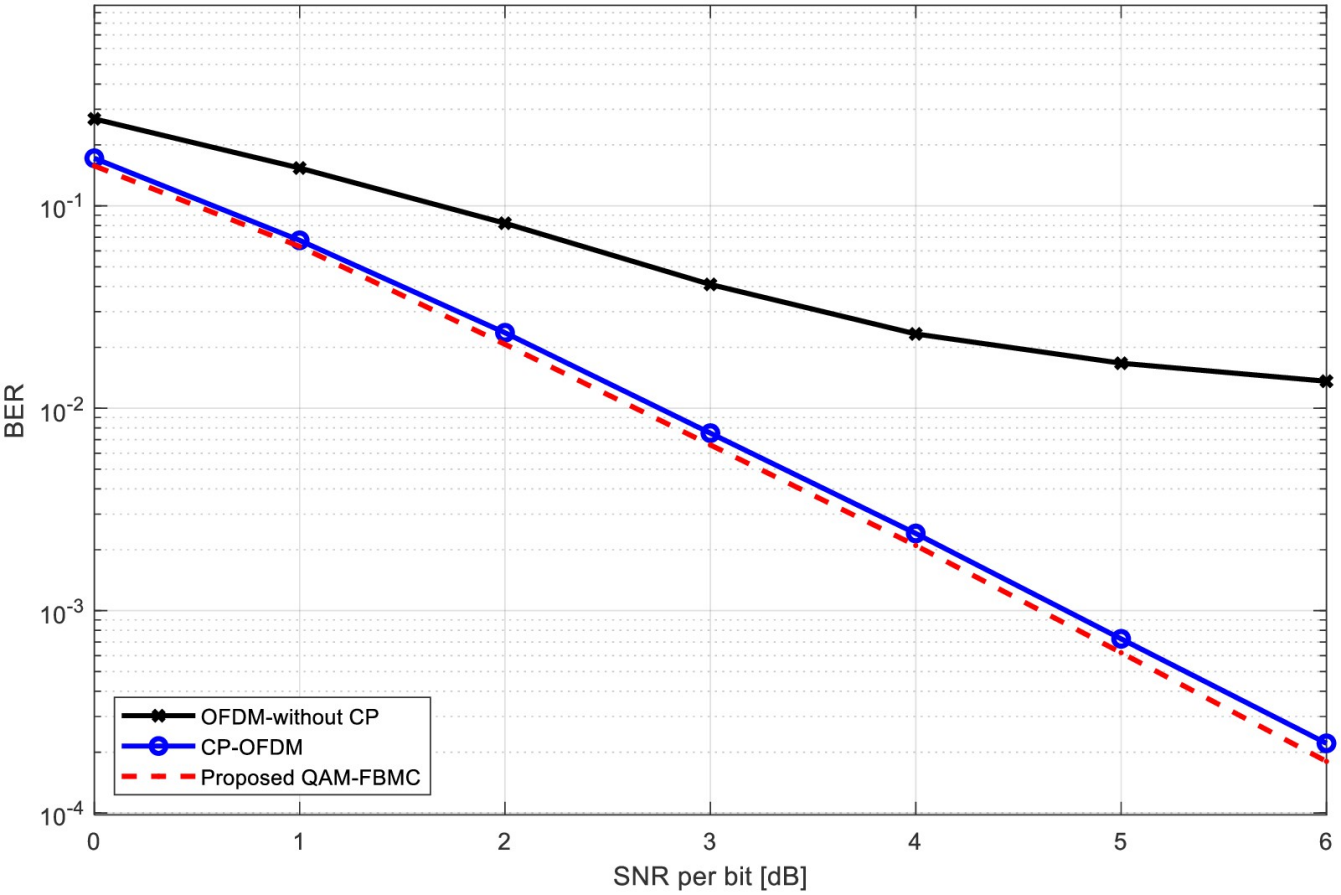
BER comparison of FBMC and OFDM with and without CP signals considering SISO configuration.

Fig 4 illustrates a comparison of BER performance among various systems, including CP-OFDM, OFDM without CP, and proposed QAM-FBMC in a frequency selective fading channel, specifically for single-input single-output (SISO) systems. This figure presents BER in terms of average signal-to-noise ratio (SNR) per bit *i*.*e*. (*E*_*b*_/*N*_*0*_). The average SNR per bit is defined as the ratio between SNR and number of transmitted bits [8]. It has been shown in [8] that the minimum average SNR per bit for error-free transmission over any type of channel is identical to that of Additive White Gaussian Noise (AWGN) channel and is equal to -1.6 dB. It’s noticeable from this figure that, the OFDM without CP exhibits the poorest BER performance when contrasted with the QAM-FBMC and CP-OFDM systems. This discrepancy due to ISI introduced by the fading channel negatively impacts OFDM without CP performance. However, it’s essential to acknowledge that implementing CP reduces spectral efficiency. For example, at a SNR per bit of 4 dB, a BER of 2×10^−3^ and 4×10^−2^ are attained for the proposed QAM-FBMC and OFDM without CP systems, respectively.

**Fig 5:**
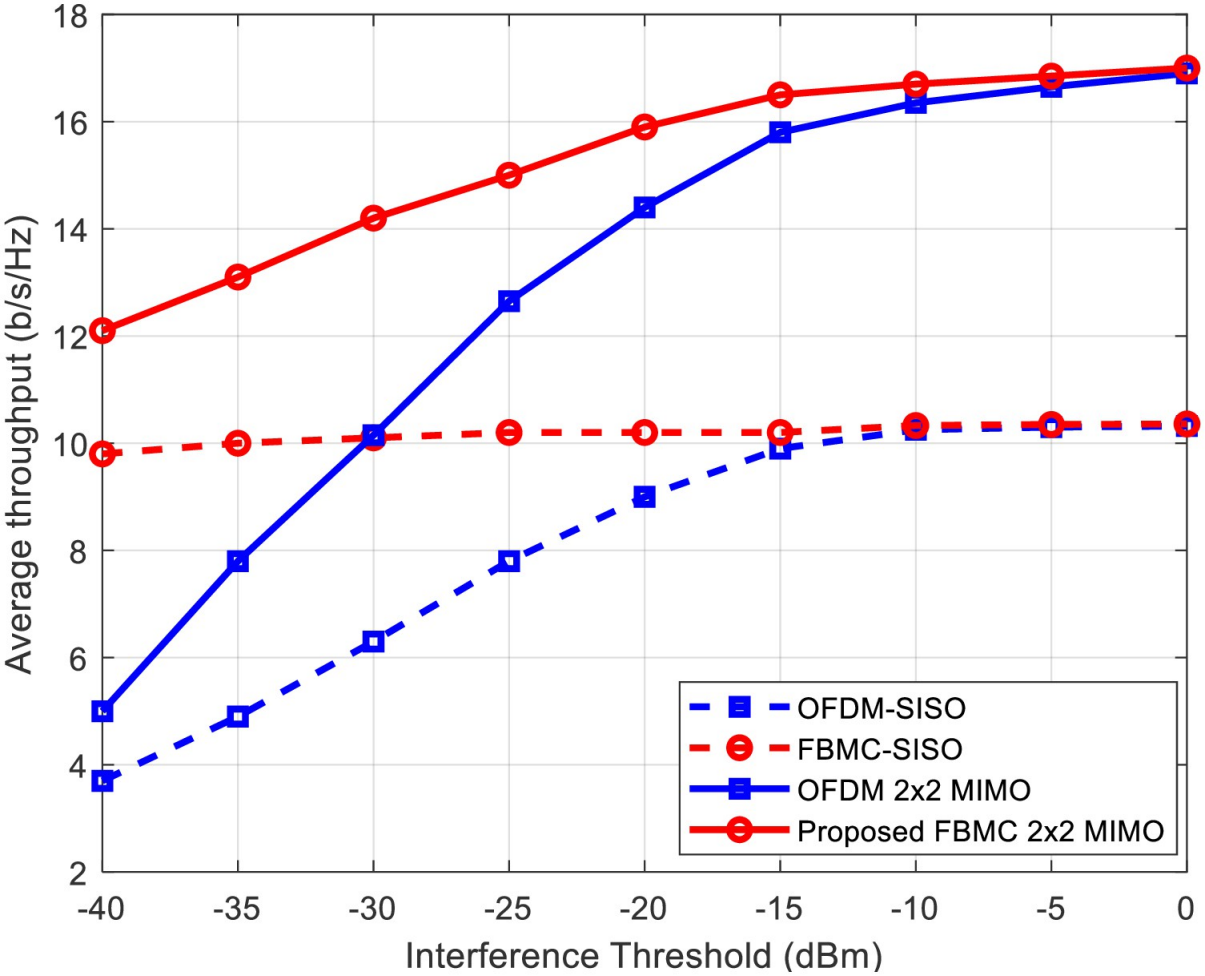
Comparing throughput between FBMC and OFDM in both SISO and MIMO configurations under different interference threshold constraints.

In Fig 5, we illustrate the average throughput of OFDM and FBMC systems for both MIMO and SISO configurations under varying interference thresholds. Several key observations can be made from this figure. First and foremost, it is evident once more that FBMC-based systems exhibit higher capacities compared to their OFDM counterparts. This underscores the advantages offered by FBMC in terms of spectral efficiency and interference mitigation. Secondly, our proposed MIMO algorithm demonstrates its effectiveness as it consistently achieves higher capacities when compared to SISO communication within both OFDM and FBMC systems. This highlights the significant performance gains made possible by MIMO technology. For example, at an interference threshold of -25 dBm, the proposed FBMC achieves more than 2 b/s/Hz higher throughput than OFDM considering 2×2 antenna configuration.

**Fig 6:**
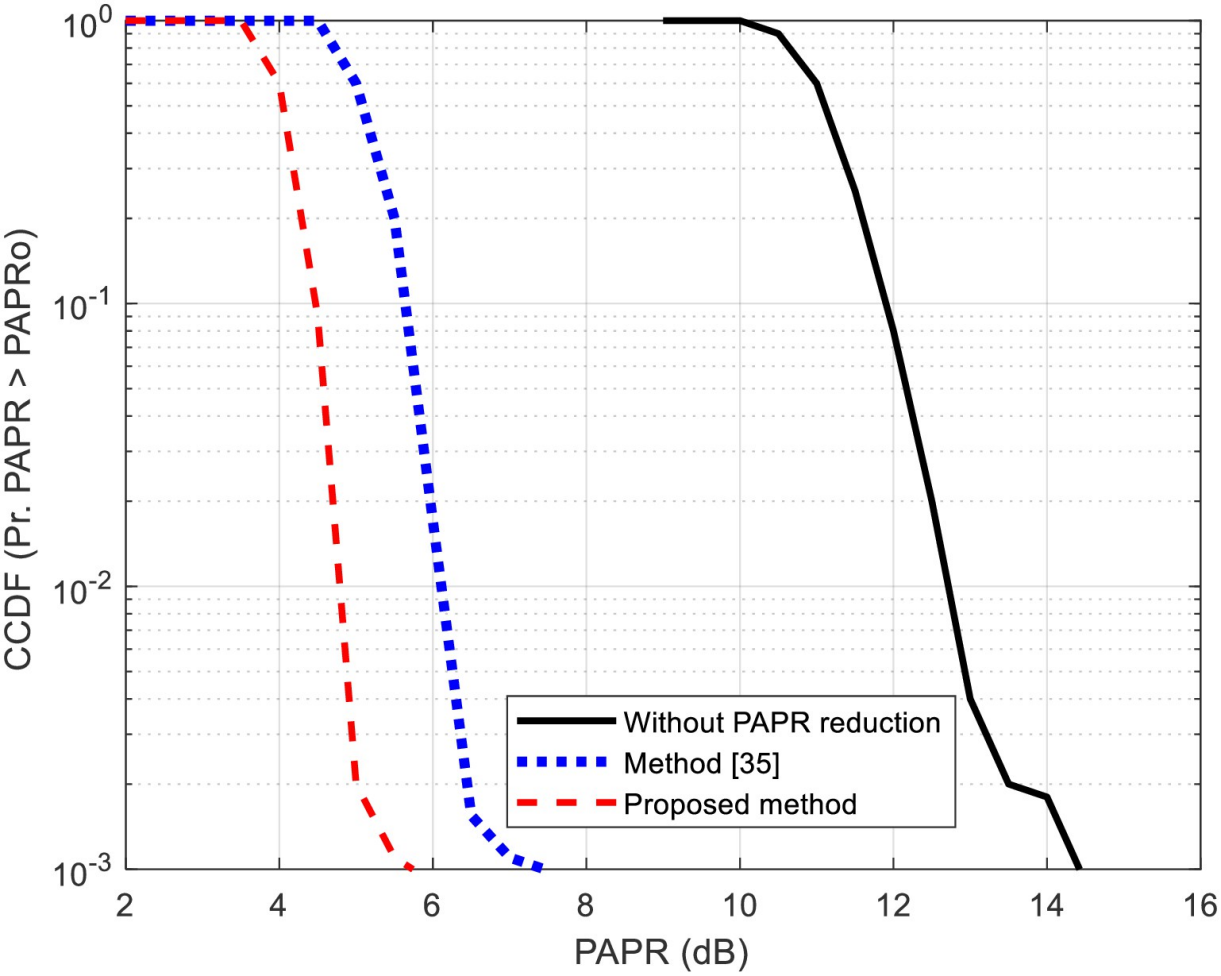
CCDF of proposed method, method in [35] and without PAPR reduction.

Fig 6 illustrates the CCDF of PAPR when the proposed PAPR reduction method is applied. To provide a comparison, the CCDF of PAPR without PAPR reduction and clipping and filtering methods in [35] are also presented. T_h_ is configured at 3 dB, and *F* is chosen as 8. The figure reveals that the PAPR levels is concurrently lowered by the proposed method. As an instance, at a CCDF of 10^−2^, the proposed method attains a PAPR reduction of 1 dB and 7.5 dB reduction in PAPR when compared to the method in [35] and without PAPR reduction, respectively.

**Fig 7:**
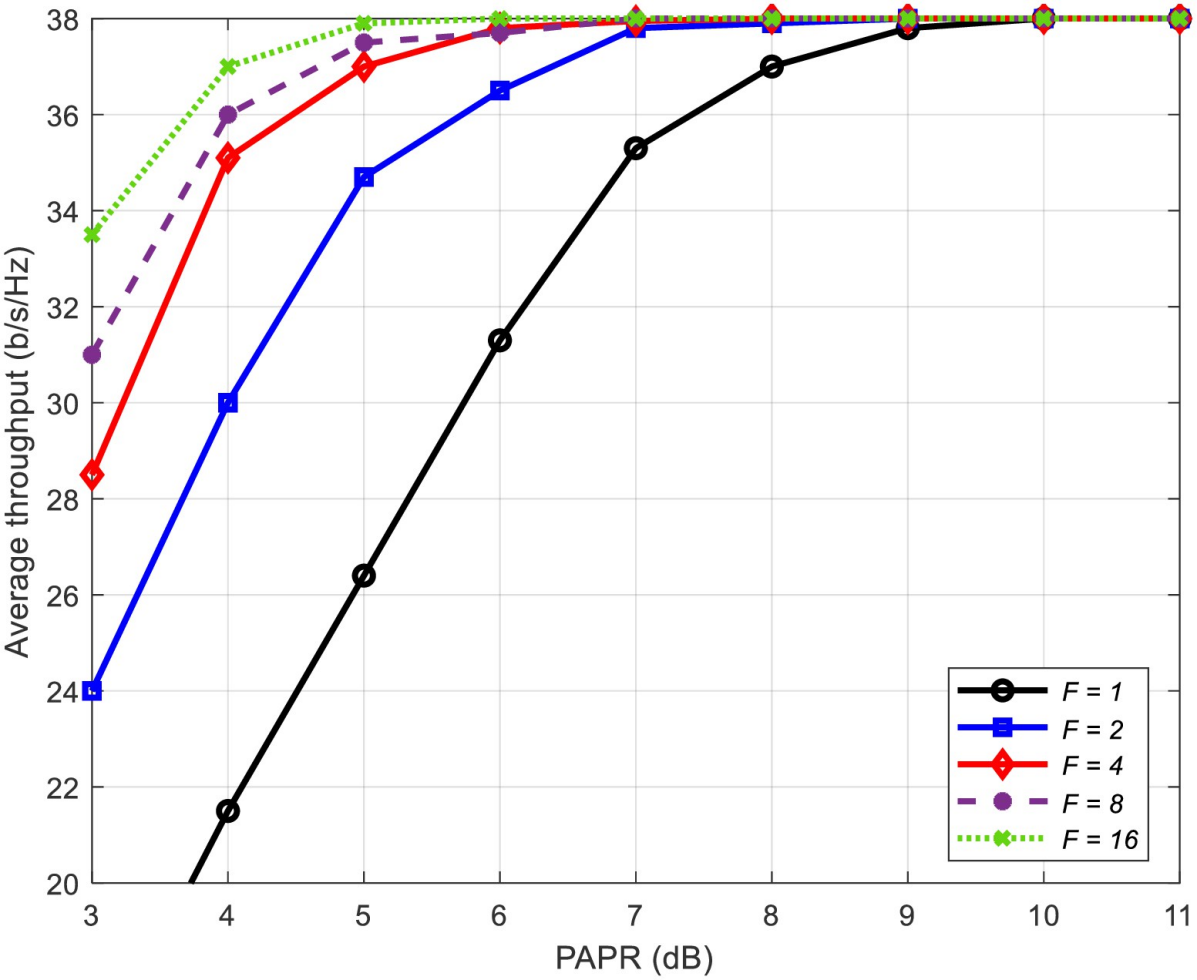
Average throughput versus PAPR using different number of frequency blocks *F*.

Fig 7 presents the average throughput as a function of the PAPR for the proposed method, considering the number of frequency blocks *F* as a variable. As *F* increases, the PAPR decrease, leading to an augmented throughput. This phenomenon can be attributed to the PAPR reduction signal component found in the channel’s null space, which tends to grow in proportion to the channel’s increasing frequency selectivity. This, in turn, aids in reducing IBO and mitigating nonlinear distortion by lowering the PAPR of the power amplifier’s input signal, all while suppressing interference to the data stream during the PAPR reduction process.

**Fig 8:**
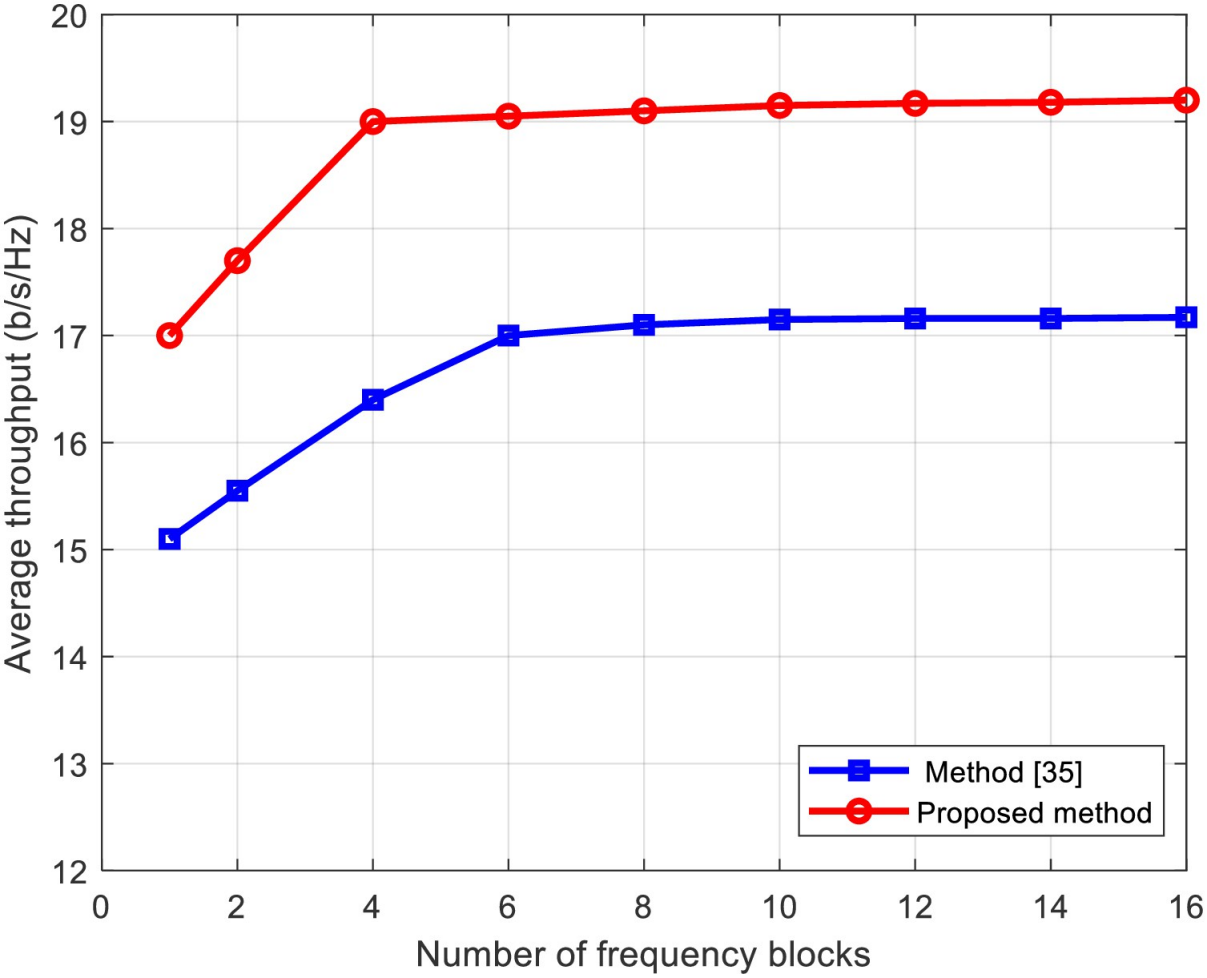
Average throughput versus number of frequency blocks for proposed method and method in [35].

**Fig 9:**
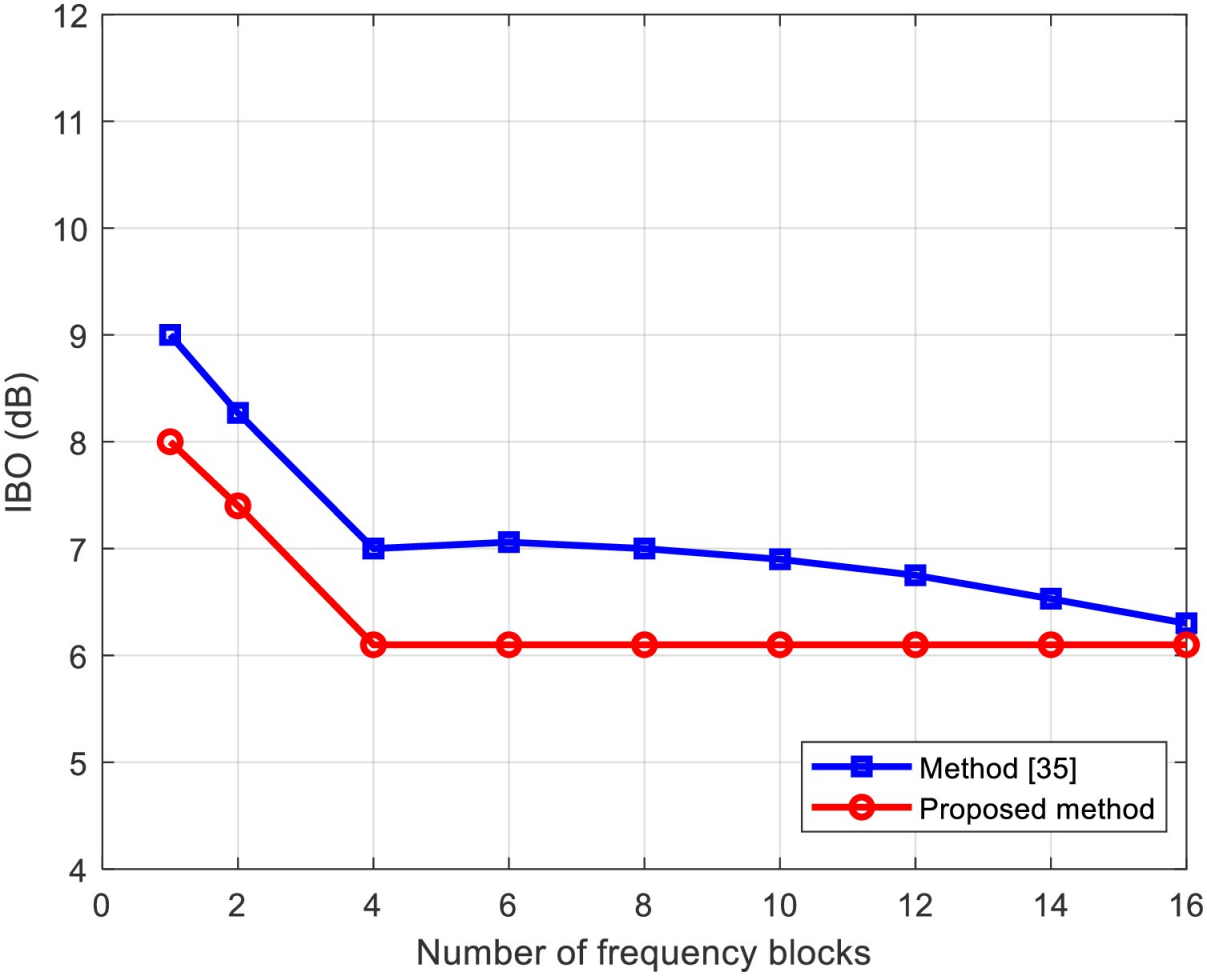
IBO versus number of frequency blocks for proposed method and method in [35].

Figs 8 and 9 present the average throughput and IBO in relation to the number of frequency blocks *F*, respectively. In addition to the proposed method, we also include the PAPR reduction technique applying clipping and filtering [35] for comparative analysis. Irrespective of the PAPR reduction method employed, as the number of frequency blocks *F* increases, signifying greater frequency selectivity in the channel, the throughput rises, and the IBO in the nonlinear amplifier decreases. This behavior is attributed to the heightened presence of the PAPR reduction signal component within the channel’s null space due to increased frequency selectivity. The proposed method demonstrates improved throughput compared to the clipping and filtering method. This improvement arises from their ability to reduce IBO more effectively than the CF method by leveraging the channel’s null space to suppress interference to the data stream caused by the PAPR reduction signal.

**Fig 10:**
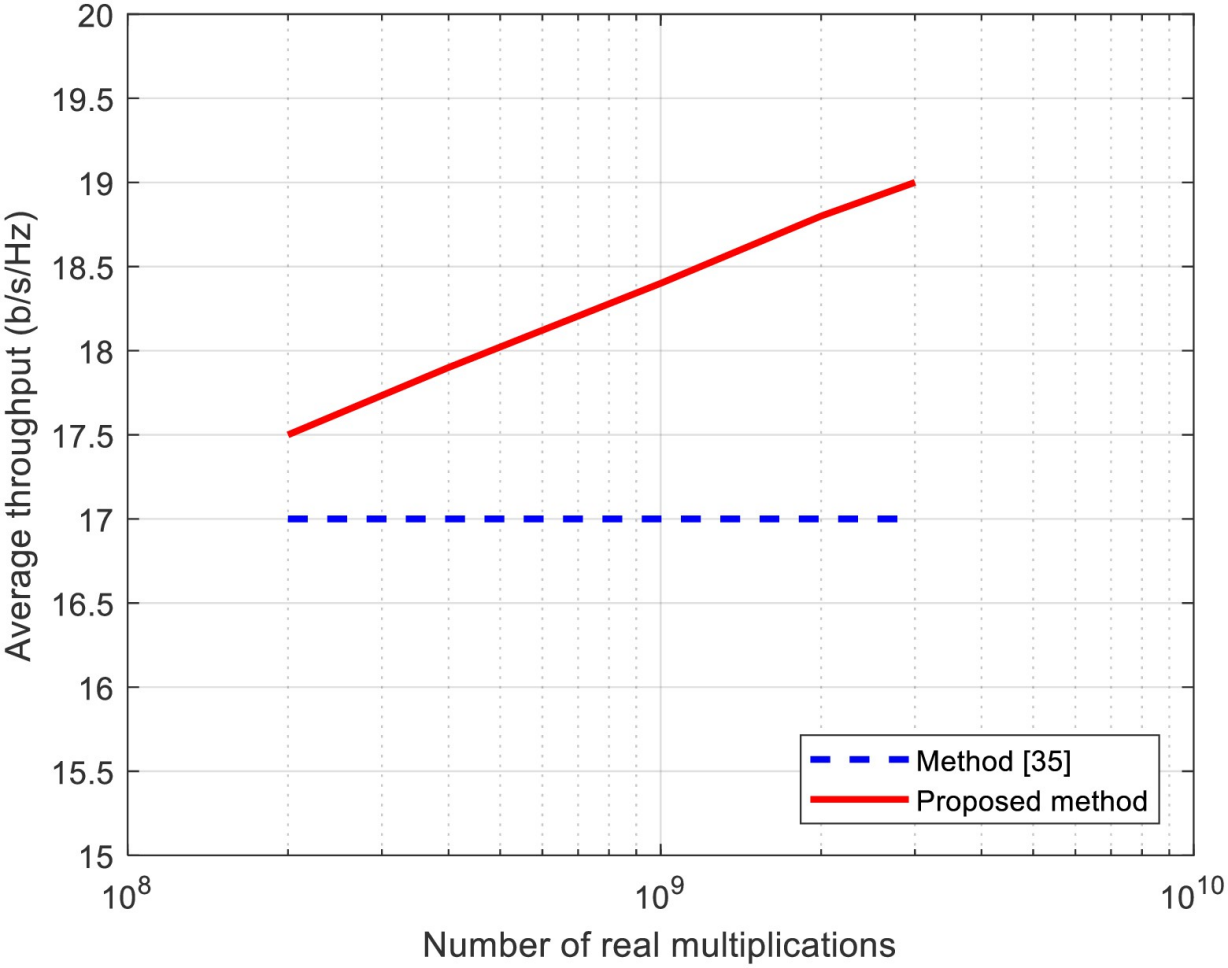
Average throughput versus no. of real multiplications for proposed method and method in [35].

Referring to the data in Table 2, Fig 10 presents the throughput in terms of the number of real multiplications for the proposed method and the CF method. In general, the throughput levels for the proposed method increases as the number of real multiplications in the PAPR reduction signal processing rises. This phenomenon occurs because with a greater number of iterations in the PAPR reduction process, the PAPR values are reduced sufficiently, resulting in smaller IBO.

**Table 2:** Number of real multiplications for the considered methods.

Method	Process Name	No. of real multiplications
**Proposed method**	Generating inverse of channel matrix	8*N*^2^*F*
	Calculation of null space matrix	4*N*^2^ (*N* − *N*_*u*_)
	IDFT	4*LNM*log_2_*F*
	Clipping	3*LNM*
	DFT	4*LNM*log_2_*M*
	Projection onto null space	4*LN*^2^*M*
	Measurement of Transmission Signal Power	2*LNM*
**CF method [35]**	IDFT	4*LNM*log_2_*M*
	Measurement of Transmission Signal Power	2*LNM*
	Clipping	3*LNM*
	DFT	4*LNM*log_2_*M*

Conversely, the throughput level for the CF method [35] experiences only marginal growth as the no. of real multiplications in the PAPR reduction signal processing increases. This is primarily due to the fact that the CF method necessitates fewer real number multiplications per iteration, allowing it to conduct more iterations compared to the method using the channel’s null space with the same no. of real multiplications. Moreover, the PAPR reduction effect per iteration in the CF method is substantial. However, the CF method introduces interference to UE receiver, limiting the enhancement of throughput with an increased number of iterations. The aforementioned outcomes underscore the importance of mitigating PAPR while simultaneously suppressing interference to UE resulting from PAPR reduction signals. This strategy facilitates higher throughput by increasing the no. of iterations in the PAPR reduction process.

## 6. Conclusion

This paper has addressed the burgeoning interest in MIMO based FBMC system as a promising multi-carrier modulation technique for next-generation communication systems. FBMC, with its manifold advantages over OFDM, has the potential to revolutionize the way we approach modern wireless communication. Nonetheless, like many MCM techniques, FBMC faces the challenge of large PAPR, which can severely impact system performance. Therefore, we have introduced a new PAPR reduction method that relies on the null space within the comprehensive channel of the entire system, which exhibits frequency selectivity. This method harnesses the null space within the MIMO channel to effectively alleviate the burden of PAPR, thereby minimizing the IBO required by nonlinear power amplifiers. The PAPR reduction signals generated through our method are strategically mapped to the null space of the integrated MIMO channel for each frequency block. The proposed method accomplishes PAPR reduction and effectively suppresses interference that may arise from the PAPR reduction signals impacting the data stream. Through computer simulations employing SSPA model, our method has proven to be successful in reducing PAPR, resulting in enhanced throughput. The performance of the proposed method surpasses that of other methods across various scenarios, regardless of factors such as the number of frequency blocks.
