# Correction: Performance enhancement and PAPR reduction for MIMO based QAM-FBMC systems

**DOI:** 10.1371/journal.pone.0341375

**Published:** 2026-01-22

**Authors:** Emad S. Hassan

The Data Availability statement for this article is incorrect. The correct statement is: The original underlying data to support all results in the article and Supporting Information files are available at Figshare (https://figshare.com/s/b72383291f366bdee1eb).

The author also states that the AI tool Grammarly was used for grammar checks during the preparation of the manuscript.

## References

[1] Hassan ES. Performance enhancement and PAPR reduction for MIMO based QAM-FBMC systems. PLoS One. 2024;19(1):e0296999. doi: 10.1371/journal.pone.0296999 38206931 PMC10783751

